# Sjogren’s Disease and Elevated Cardiovascular Risk: Mechanisms and Treatment

**DOI:** 10.3390/jcdd12090367

**Published:** 2025-09-18

**Authors:** Jennifer Behbodikhah, Billy Ding, Belin Jacob, Nuzhat Batool, Elise Belilos, Joshua De Leon, Steven E. Carsons, Allison B. Reiss

**Affiliations:** 1 Department of Medicine, NYU Grossman Long Island School of Medicine, Mineola, NY 11501, USA; jennifer.behbodikhah@gmail.com (J.B.); billy.ding@nyulangone.org (B.D.); berlin.jacob@nyulangone.org (B.J.); nuzhat.batool@nyulangone.org (N.B.); elise.belilos@nyulangone.org (E.B.); joshua.deleon@nyulangone.org (J.D.L.); 2 Department of Foundations of Medicine, NYU Grossman Long Island School of Medicine, Mineola, NY 11501, USA

**Keywords:** Sjögren’s disease, cardiovascular risk, atherosclerosis, autoimmune disorder, dyslipidemia, treatment

## Abstract

Autoimmune disorders are known to accelerate atherosclerosis, increasing the rate of cardiovascular disease. As the number one cause of morbidity and mortality in the general population, this risk is only enhanced in inflammatory conditions. Substantial evidence links increased cardiovascular disease to systemic lupus erythematosus and rheumatoid arthritis. However, Sjogren’s Disease (SjD) tends to follow a more indolent disease course, and its chronic inflammatory burden is often underrecognized. Pharmacologic agents are also limited and symptom management is often the mainstay of treatment. The majority of studies investigating cardiovascular disease in SjD show conflicting results. In this review, we shed some light on the association of SjD and cardiovascular disease. Furthermore, we also explore potential risk factors and mechanisms through which SjD may accelerate cardiovascular disease. We address the impact of standard CVD and SjD treatments on heart health and highlight clinically relevant tools for monitoring subclinical atherosclerosis in the SjD patient population.

## 1. Introduction

Cardiovascular disease (CVD) has long been known as a leading cause of morbidity and mortality in many autoimmune rheumatic disorders [1,2]. Chronic inflammation plays a key role in the mechanism of atherosclerotic damage and has been shown to lead to premature cardiovascular events [3].

Sjogren’s disease (SjD) is a systemic chronic autoimmune disorder that involves immune-mediated damage of the salivary and lacrimal glands that ultimately leads to dryness of the mouth (xerostomia) and eyes (xerophthalmia) [4]. Extraglandular effects are found in between 30 and 40% of patients due to immune complex deposition and lymphocytic infiltration in the kidney, lung, central nervous system and other organs [5,6]. With a variable prevalence rate between 13.1 and 60.8 per 100,000 inhabitants, SjD is one of the most common autoimmune diseases. Similarly to many other rheumatic conditions, SjD tends to primarily affect the female population, influencing mostly middle-aged Caucasian women, with a female to male ratio between 6 and 10.7 [7,8,9].

Like most autoimmune disorders, the cause of SjD is unknown, but the inflammatory state associated with the condition has long been thought to place these patients at risk for other comorbidities [10]. CVD risk factors have been well recognized in the initiation and progression of atherosclerosis. These risk factors, such as diabetes, hypertension, smoking, hyperlipidemia and family history, have a strong association with cardiovascular events [11]. In states of chronic inflammation, studies have shown an acceleration of atherosclerotic damage [12]. This process is thought to be further enhanced by lack of exercise in debilitating systemic illness, atherogenic medications, sedentary lifestyles, and other contributing factors [13]. The acceleration of atherosclerotic disease has been well studied in systemic lupus erythematosus (SLE) and rheumatoid arthritis (RA), and the management and evaluation have been thought to be similar in SjD. However, SjD frequently follows a more benign course, and patients often go without any immunosuppressive therapy despite chronic inflammation. Therefore, the degree of atherosclerotic damage is likely overlooked in this subset of patients [14,15].

This review aims to clarify the association of SjD and CVD. Furthermore, we plan to focus on the clinically relevant literature to provide clinicians with insights into navigating the intertwined pathophysiology of these two conditions.

## 2. Epidemiology

### 2.1. Relative Risk of CVD in Patients with SjD

According to the 2019 American Heart Association (AHA) report, approximately 48% of U.S. adults are affected by CVD, including coronary artery disease, stroke, heart failure, and hypertension [16]. CVD remains the leading cause of morbidity and mortality in the United States, accounting for approximately one in five deaths in 2022 [17]. Established modifiable (e.g., hypertension, diabetes, dyslipidemia, smoking) and nonmodifiable (e.g., age, sex, family history) cardiovascular risk factors are well recognized as predictors of cardiovascular events in the general population [18,19]. These risk factors promote endothelial dysfunction, increase carotid intima-media thickness (IMT), and accelerate atherosclerotic plaque formation [20,21,22]. Their impact is exacerbated in the context of chronic autoimmune and inflammatory diseases, due to persistent systemic inflammation and immune dysregulation [23].

The increased risk of CVD in SLE and RA has been well established, with both conditions associated with a higher incidence of myocardial infarction and stroke [24,25]. Both SLE and RA are now recognized as independent risk factors for atherosclerosis, where disease mechanisms involve pro-inflammatory cytokines (e.g., tumor necrosis factor (TNF)-α, interleukin (IL)-6, type 1 interferons), oxidative stress, and endothelial dysfunction, which in turn lead to fibrosis of the vasculature and proliferation of smooth muscle cells [26,27,28,29,30,31]. Investigations of these events are confounded by the use of long-term pharmacologic agents that may alter and even enhance the atherosclerotic effects. However, SjD tends to have a more benign course without the use of significant immunosuppressive drugs and other agents [32]. Multiple studies have reported on the risk of cardiovascular events in SjD, but the studies have conflicting results.

Early prospective work by Theander et al. found only a modest increase in cardiovascular mortality among patients with SjD, with a standardized mortality ratio of 1.06 in cardiovascular-related death in a group of 265 SjD patients being followed over the span of 7 years [33]. However, recent data suggests a more significant mortality risk in this population. Rusinovich et al. looked at mortality in a cohort of 314 SjD patients and found that the mortality rate was 70% higher compared to the general population, with CVD ranking as the third leading cause of death and accounting for 7.1% of deaths in this group [34]. These findings indicate that while earlier studies may have underestimated the risk, there is accumulating evidence that points towards SjD as a major contributor to cardiovascular mortality.

A 2015 study by Bartoloni et al., provided insight into cardiovascular risk in SjD through a retrospective analysis of cardiovascular risk factors and events in 1343 patients [35]. Their study found that hypercholesterolemia and hypertension were more commonly found in these patients, as opposed to diabetes, obesity and smoking. More importantly, cerebrovascular events (2.5% vs. 1.4%, *p* = 0.005) and myocardial infarction (1.0% vs. 0.4%, *p* = 0.002) were more common in patients with SjD as opposed to the control group. Additionally, older age and longer disease duration were significantly associated with an increased prevalence of cardiovascular events (*p* ≤ 0.04). These findings suggest that the pattern of risk in SjD may differ from the traditional pattern seen in the general population. On the other hand, a large cohort study by Chiang et al. oversaw 5205 patients with SjD from 2000 until 2006 and did not find a statistically significant difference in the incidence of acute myocardial infarction [36]. The discrepancies in these studies highlight the complexity of cardiovascular risk assessment in SjD and suggest that differences in population, study design and disease heterogeneity may influence study outcomes.

Ankle-brachial-index (ABI) is generally a low-cost and reliable test for detecting peripheral artery disease and therefore is often utilized as a predictor of cardiovascular disease [37]. Rachapalli et al. measured ABI in a small group of patients with SjD and disease duration greater than 5 years [38]. Although there was a higher prevalence of patients with ABI less than 1 in the SjD group, the results were not found to be statistically significant, perhaps because of the sample size. However, a statistically significant association was found between decreased ABI and disease duration, further supporting the increased risk of CVD with greater disease duration and chronic inflammation.

Evidence of atherosclerosis in SjD has also been demonstrated through vascular imaging. Vaudo et al. studied carotid and femoral artery intima-media thickness in a small group of women with SjD at the University of Perugia [39]. Patients in the subject group were found to have a higher intima-media thickness than the control group in both the carotid artery (mean SD 0.82 ± 0.24 mm versus 0.63 ± 0.20 mm; *p* ≤ 0.001) and femoral artery (SD 0.81 ± 0.26 mm versus 0.67 ± 0.23 mm; *p* ≤ 0.019). Similar results have been found in RA and SLE patients, however this study is unique in that it represents a young, female population, generally protected from atherosclerosis. Furthermore, this group of patients represents those who are not on long term therapies generally used to control inflammatory disease and thereby removing a confounding factor in other investigations of CVD.

Given the rising number of observational studies showing an increased risk of cardiovascular events in SjD, Yi et al. sought to determine if there is a genetic association between SjD and the risk of ischemic heart disease and stroke [40,41]. Their primary objective was to detect pleiotropic genes that may predispose this patient population to cardiovascular events. Using two sample and multivariable Mendelian randomization, they found a strong genetic link between the SjD and CVD further supporting this theory. In addition to reinforcing epidemiologic findings, their work also identified key genes that have the potential to be used as molecular targets for therapeutic intervention in this population.

Furthermore, Loiseau et al. conducted a cohort study in Denmark to evaluate cardiovascular disease outcomes among patients newly diagnosed with SjD between 1996 and 2017. The outcomes measured included first time myocardial infarction, stroke, peripheral artery disease, thromboembolism, heart failure and cardiovascular mortality (within 30 days of the cardiovascular event) in SjD [42]. In this study, 4967 patients were evaluated and followed for at least one year after diagnosis. Patients with presumed secondary Sjogren’s were excluded from the study, as were patients with prior hospitalizations for cardiovascular related events. At baseline, patients with SjD exhibited elevated rates of cardiovascular risk factors at baseline, such as diabetes and obesity, and increased use of pharmacologic treatments, such as glucocorticoids, NSAIDs and immunosuppressive agents. Over the follow-up period, SjD was found to be associated with MI (adjusted hazard ratio [aHR] 1.23; 95% confidence interval [CI] 1.01–1.50), ischemic stroke (aHR 1.31; 95% CI 1.14–1.52), hemorrhagic stroke (aHR 1.51; 95% CI 1.13–2.03), PAD (aHR 1.44; 95% CI 1.13–1.83), and venous thromboembolism (aHR 1.57; 95% CI 1.33–1.85). These findings provide evidence that SjD is an independent risk factor for various cardiovascular events.

### 2.2. Traditional Cardiovascular Risk Factors

Traditional cardiovascular risk factors, such as obesity, hypertension, hyperlipidemia, smoking and family history are generally thought to be accelerated in the chronic inflammatory state characteristic of autoimmune diseases [43]. Most of the research on these effects focuses on SLE and RA, while less is known about the relationship between these risk factors and SjD. This can be attributed to the relatively low prevalence of SjD and the many shared risk factors present in both CVD and SjD. Shared risk factors lead to confounding bias that can obscure the true association.

Obesity, classified by the World Health Organization and the Centers for Disease Control as a BMI of 30 and above, is one of the most common health conditions in the world [44,45]. Adipose tissue in obesity is thought to secrete substances that induce and sustain a chronic inflammatory state that accelerates CVD [46,47]. Lipid profile abnormalities also lead to accelerated atherosclerosis and these alterations in lipid profile pattern are due partially to inflammatory triggers as well as to effects of commonly used medications in autoimmune disease. In a study by Mezei et al., features of obese patients with SjD were compared to those without a history of obesity [48]. Interestingly, while no difference was found in cholesterol levels between the two groups, triglycerides were found to be higher in the obese population. Anti-Ro (SSA) and anti-La (SSB) antibodies as well as rheumatoid factor were less commonly positive in the obese group. Based on their observations, obese patients required steroids less often and were more commonly on statin therapy for hyperlipidemia. More importantly, disease activity appeared to be lower in the obese cohort, with a median EULAR-SS Disease Activity Index (ESSDAI) in the obese group of 2 (0–3, n = 61), compared to 4 (2–7, n = 38) (*p* < 0.001) in the non-obese group. These results suggest a rather surprising protective effect of obesity in patients with SjD and raise important questions regarding the relationship between immune activation and adiposity.

In SjD, glucocorticoids tend to be used to treat extraglandular manifestations, such as arthralgias and cutaneous disease. When glucocorticoids are insufficient, disease modifying anti-rheumatic drugs (DMARDs) tend to be added on to the regimen. The results of the study by Mezei et al. showed that steroids and DMARDs were less commonly used in the obese population, further suggesting a mild disease course in these patients [48]. Furthermore, statins have known anti–inflammatory effects [49]. Immune cells are normally found within atherosclerotic plaques. By blocking specific intracellular pathways, statins can decrease the number of inflammatory cells, reduce T cell activation and prevent the migration of inflammatory cells into plaques. In this study, obese patients were more likely to be on statin therapy. Additionally, this group of patients had a significantly lower ESSDAI score, suggesting a beneficial role of statin therapy in SjD and possibly other rheumatic diseases [50,51,52,53,54]. Statin therapy has been studied in other autoimmune diseases as well. In persons with RA, statins decrease mortality, lower DAS28 scores, swollen joint count and stiffness [55,56,57,58]. Discontinuation of the medication has also been shown to increase mortality risk in RA [59]. These findings point to statins as a potentially valuable adjunctive therapy to autoimmune diseases such as SjD, where they may address both cardiovascular risk and disease activity.

Among traditional cardiovascular risk factors, hypertension tends to have the highest incidence in chronic inflammatory diseases and is the most important factor in propagating atherosclerosis [60]. Prior studies have shown a strong association between hypertension and autoimmune conditions such as lupus and RA [61]. This strong relationship can be, in part, explained by shared inflammatory mechanisms. Hypertension induces arterial wall injury, which activates cytokines and recruits inflammatory cells. Some studies have even shown that autoantibodies in lupus are associated with hypertension, as is an increase in immunoglobulins suggesting more of an immune mediated link between the two than was previously unknown [62].

Smoking, a well-established modifiable cardiovascular risk factor, has complex interactions with SjD. In patients with SjD, smoking has been associated with decreased high-density lipoprotein (HDL) and increased rates of hypertension [63,64]. However, the relationship between the development of SjD and smoking appears paradoxical. A recent systematic review by Bandeira et al. of approximately 15 articles found that active smoking is associated with a lower risk of developing SjD [65]. However, the results on past smoking were controversial. Bandeira et al. also sought to find an association between the presence of autoantibodies and smoking, however studies showed variable results similar to the association with complaints of dryness. An analysis of a prospective collection of data on 300 patients fulfilling the Copenhagen Classification Criteria for SjD was utilized by Manthorpe et al. to assess the association between FOCUS scores on lip biopsy and smoking habits [66]. They found that tobacco tends to decrease FOCUS scores in a dose dependent manner suggesting a negative correlation between smoking and SjD diagnosis. They found a similar pattern in the presence of anti-SSA/RO and anti-SSB/La. These findings highlight the importance of the need for other objective data in this patient population to avoid missed diagnoses. Furthermore, a few studies have also hypothesized that smoking may only play a minor role in increasing cardiovascular risk in women with SjD, highlighting the need for nuanced assessment of traditional risk factors in this population [67,68].

### 2.3. Risk Factors Specific to SjD

Chronic inflammation plays a critical role in the acceleration of atherosclerosis [69]. C-reactive protein (CRP) has been well studied in association with cardiovascular risk and is commonly used in practice by rheumatologists as a marker of inflammation in numerous connective tissue disorders [70,71]. In a study by Vaudo et al., CRP levels were not found to differ between the SjD group and the healthy control group, perhaps implying a low grade of inflammation [39]. Moreover, CRP also was not found to be associated with intima-media thickening in this study or in a study by Ozisler [72]. This suggests that there may be a noninflammatory factor involved in the pathogenesis of atherosclerosis within this subset of patients. The Vaudo study showed lower levels of HDL and triglycerides amongst patients with SjD in comparison to the control group, representing an altered lipid profile in SjD, while no difference was found by Rachapalli et al. when studying ABI [38,39]. In line with the established relationship between inflammation and cardiovascular risk, a retrospective case–control study of a cohort of patients with a diagnosis of SjD, those with 3 or more CVD risk factors had a higher mean CRP level, underscoring the interplay between cumulative risk factors and systemic inflammation [73].

When Vaudo et al. studied carotid and femoral artery intima-media thickness in a group of women with SjD, they also found that patients in the subset with a higher carotid IMT also showed a higher prevalence of leukopenia and circulating anti-SSA antibodies [39]. In SjD, a low white blood cell count is associated with a greater degree of salivary gland inflammation. Anti-SSA antibodies are thought to induce leukopenia via apoptotic events. Furthermore, apoptosis plays a key role in the cascade of atherosclerosis development. In prior studies, augmented apoptosis in lupus has been hypothesized to accelerate atherosclerosis, suggesting a similar mechanism in SjD [74,75,76,77,78]. These findings raise the possibility that similar apoptotic events may explain the increased atherosclerotic risk observed in SjD.

Table 1 provides a summary of the elements of CVD risk to be considered in persons with SjD.

## 3. Pathophysiology and Mechanism

### 3.1. Cytokine (Interleukin and Interferon)

The close interplay between cytokines and immune cells is central in the pathophysiology of SjD, a disorder characterized by lymphocytic infiltration of exocrine glands. Elevated levels of IL-17 are seen in the plasma and salivary gland biopsies of patients with SjD [79,80]. In addition, immunohistochemical studies of salivary gland specimens also demonstrated increased levels of TGF-β. Both IL-17 and TGF-β positively correlated with disease activity and advanced disease. Activated epithelial cells within affected salivary glands produce and secrete IL-7, IL-18, and IL-22, which are key mediators involved in the adaptive immune response and T cell activation, promoting CD4 T cells to secrete IL-2, IL-10, and interferon (IFN)-γ. This perpetuates systemic chronic inflammation and inflammation within salivary glands [81,82]. More recently, calprotectin, a complex of S100A8 and S100A9 proteins highly expressed within the cytoplasm of neutrophils, emerged as a possible marker for atherosclerosis in patients with SjD. It serves as a key pro-inflammatory mediator of innate immunity, activating toll-like receptor (TLR)-4 by acting as an endogenous damage-associated molecular pattern molecule. In patients with SjD, increased levels of calprotectin were found not only at the glandular site but also within the circulation. Its pro-inflammatory effect on endothelial cells promotes the production of other inflammatory mediators, such as IL-1β, IL-6, TNF-α, IFN-γ, IL-10, IL-17A, and IL-22, thereby linking activation of the innate immune system to vascular pathology in SjD [83,84].

Bartoloni et al. were also able to demonstrate other markers of endothelial activation that are increased in patients with SjD compared to control, including soluble thrombomodulin (sTM) and plasma asymmetric dimethylarginine (ADMA). Thrombomodulin, a membrane glycoprotein receptor that binds thrombin, regulates fibrinolysis and coagulation. Its expression within atherosclerotic lesions propagates atherosclerosis via mitogenic activity in vascular smooth cells. Similarly, Luczak et al. demonstrated elevated levels of plasma ADMA in patients with SjD [85]. These levels also correlated positively with increased measures of disease activity. ADMA, a strong endogenous inhibitor of nitric oxide synthase, induces endothelial dysfunction via various mechanisms, including increased TNF-α production, heightened oxidative stress, and decreased nitric oxide production by inhibiting nitric oxide synthase, all of which can accelerate atherosclerosis [85].

Elevated circulating cytokines and pro-inflammatory markers such as soluble thrombomodulin, anti-endothelial cell antibody, intracellular adhesion molecule 1 (ICAM-1), vascular adhesion molecule 1 (VCAM-1) and asymmetric dimethylarginine drive the infiltration of monocytes, T cells, and dendritic cells into the intima, promoting atherosclerotic plaque development [86].

### 3.2. Immune Dysregulation

The abnormal activation of T and B lymphocytes is a key mediator of the inflammatory response involved in endothelial activation and injury. Liu et al. conducted a cross-sectional study that explored the correlation between immune cells and atherosclerosis in patients with SjD [87]. Interestingly, they noted a positive correlation between increased carotid IMT and levels of IgA and a negative correlation with levels of IgM. B lymphocytes, responsible for the humoral immune response and immunoglobulin production, can promote arterial inflammation, but their effects are context-dependent and the exact atherogenic/atheroprotective mechanisms of IgA over IgM remain to be elucidated. Prior studies, such as those conducted by Karvonen et al., have demonstrated an inverse correlation in the levels of circulating IgM subtypes of autoantibodies of oxidized LDL and subclinical atherosclerosis [88]. These autoantibodies promote the removal of oxidized lipoproteins within the plasma and prevent their infiltration into the arterial wall. Ankle brachial pulse wave velocity positively correlated with disease duration, levels of CRP, T helper cells, and B-lymphocytes, but negatively correlated with levels of IgM. T-helper cells alone can secrete inflammatory mediators, such as TNF-α, IL-1, and IL-6, that further endothelial injury and inflammation within the vessel walls [87]. Specifically, the subtypes of T-helper cells, Th1 and Th17, have been linked to the pathogenesis of atherosclerosis in patients with SjD. Th17, in particular, is a key producer of IL-17, a pro-inflammatory mediator that propagates chronic inflammation, salivary gland injury, and other pro-atherogenic effects. It incites not only the expression of adhesion molecules (e.g., ICAM-1) but also induces the development of fibrosis and promotes plaque instability. Similarly, Th1 cells promote the release of key cytokines, such as IFNγ, IL-2, IL-12, IL-18, and TNF-α, which play a vital role in endothelial damage and plaque formation [84,89].

### 3.3. Endothelial Activation

Endothelial activation is a central step in the pathogenesis of atherosclerosis, as it promotes the production of several adhesion molecules like ICAM-1 and VCAM-1, which are key biomarkers of endothelial injury [90]. These molecules, which are significantly upregulated in the serum of patients with SjD, facilitate immune cell migration and recruitment into the vessel wall [91]. Monocytes recruited into the subendothelial space differentiate into macrophages and take up oxidized low-density lipoprotein (LDL) via scavenger receptors leading to transformation into atherogenic foam cells [92]. This process further upregulates toll-like receptors (TLRs), amplifying local inflammation. TLR expression is increased in both mouse and human models of SjD, and the resulting immune overactivation serves as a potential link between the innate immune system and the pathogenesis of atherosclerosis. Additionally, macrophages contribute to the development of atherosclerosis by releasing reactive oxygen species and inducing oxidative stress. This in turn, promotes the formation of oxidized LDL, which furthers lipid accumulation and the formation of foam cells, further perpetuating the atherogenic cascade [15,93].

Endothelial dysfunction, an early and potentially reversible phase of vascular disease, is often used as a reliable indicator of cardiovascular mortality and morbidity in several inflammatory and autoimmune disorders. It is a key step in the onset of subclinical atherosclerosis, and several events propagate endothelial injury. For example, an imbalance between endothelial microparticle release and endothelial progenitor cell generation is a key contributor to endothelial dysfunction [94,95]. Endothelial microparticles are vesicular cell fragments that are shed from the cell surface in greater numbers with endothelial injury. Endothelial progenitor cells infiltrate areas of vascular injury and transform into mature endothelial cells that can repair the damaged arterial wall. An altered balance between microparticles and progenitor cells perpetuates disruption of the integrity of the endothelial cell monolayer and incites vascular thrombosis. Conditions such as diabetes, CVD, and systemic arterial hypertension are all associated with increased circulating microparticles [96]. Likewise, decreased capacity and number of endothelial progenitor cells are seen in these conditions, fostering an imbalance between endothelial injury and repair.

Bartoloni et al. were the first to demonstrate this imbalance in patients with SjD. They discovered a significant reduction in circulating endothelial progenitor cells and impaired reparative potential, which directly correlated with disease course in the study population [94]. Similarly, Caraba et al. investigated the association between SjD disease activity via serum biomarkers and endothelial function via flow mediated dilation of the brachial artery [97]. Compared to controls, patients with SjD had significant endothelial dysfunction as demonstrated by lower percentages of flow mediated dilation. The presence of endothelial dysfunction in patients with SjD also positively correlated with elevated levels of pro-inflammatory mediators, such as TNF-α, IL-6, and beta-2 microglobulin, further corroborating that vascular dysfunction is a key component in the pathogenesis of SjD and a potential contributor to increased cardiovascular risk.

### 3.4. Lipid Abnormalities and Lipoproteins

Compared to healthy controls, patients with SjD demonstrate a higher incidence of dyslipidemia, and those with more significant lipid abnormalities had higher inflammation levels and more robust autoimmune responses [63,98]. Lipid profiles of those with SjD were characterized by lower HDL cholesterol and elevated total cholesterol, LDL and triglycerides. Abnormal lipid profiles can influence immune cell function and promote inflammation. For example, dyslipidemia promotes lipid deposition within exocrine glands by inducing lipoprotein lipase to break down triglycerides into fatty acids. The abnormal metabolism of saturated and unsaturated fatty acids can promote mitochondrial dysfunction, causing stress in the endoplasmic reticulum and the production of reactive oxidative species [99]. This process can cause salivary and lacrimal glandular cell death. Dyslipidemia can also activate the TLR-4 pathways and promote IL-27 production, which draws immune cells to affected exocrine glands. IL-6 production within these affected glands promotes B cell maturation and plasma cell survival, which support the inflammatory environment, worsening the autoimmune response and dyslipidemia seen in patients with SjD [100].

Augusto et al. found that patients with SjD had a higher frequency of metabolic syndrome. This, in itself, puts these patients at increased risk for asymptomatic atherosclerosis and cardiovascular events [101]. Oxidized LDL plays a central role in the pathogenesis of atherosclerosis. LDL oxidation within the subendothelial space promotes macrophage infiltration, activation and the release of chemokines and adhesion molecules. Oxidized LDL activates the innate immune system via pattern recognition receptors while simultaneously serving as autoantigens in the humoral and cellular immune responses. This promotes the migration of vascular smooth muscle within the plaque, where they exhibit great plasticity and contribute to the formation of the fibrous plaque [86,102]. Cinoku et al. investigated the association of autoantibodies against oxidized LDL and the pathogenesis of arterial plaque formation and arterial wall thickening in patients with RA, SLE, and SjD [103]. Although no clear association was found between the two factors in patients with RA or SLE, in patients with SjD, anti-oxidized LDL antibodies served as an independent protective factor against arterial plaque formation after correcting for potential confounders. The exact mechanisms behind this association have yet to be elucidated. Still, potential explanations include increased clearance of oxidized LDL from circulation and IgM-specific anti- oxidized LDL antibodies’ ability to block macrophage uptake of oxidized LDL via scavenger receptors, preventing foam cells from forming. Similarly, Yang et al. conducted a retrospective study in China that involved 367 patients with SjD to investigate the relationship between lipid profiles and disease activity [63]. Elevated inflammatory markers such as erythrocyte sedimentation rate (ESR) and increased autoimmunity were associated with more severe dyslipidemia. Other non-traditional cardiovascular risk factors such as decreased complement also contributed to increased hyperlipidemia-associated complications in patients with SjD. It is valuable to take into account the interplay between metabolic dysregulation and chronic inflammation in devising ways to minimize the cardiovascular risks associated with SjD.

### 3.5. Vessel Abnormalities

Endothelial dysfunction, an early insult in the pathogenesis of atherosclerosis, is prevalent among SjD patients, making them highly susceptible to developing atherosclerosis. Several studies have tried to elucidate the effect of chronic inflammation on vessel abnormalities. In one study, Vaudo et al. found higher intima-media thickening in the carotid and femoral arteries among 37 patients affected by SjD compared to 35 healthy controls [39]. This study demonstrated that anti-SSA antibodies independently correlated with arterial thickening. In a larger assessment, Karakasis et al. evaluated the risk of subclinical atherosclerosis in patients with SjD using markers such as carotid and femoral intima-media thickness and pulse wave velocity [104]. 19 observational studies encompassing 1625 patients demonstrated that when compared to controls patients with SjD had higher carotid and femoral IMT. In addition, elevated ESR and a longer disease duration were positively associated with higher IMT, while measures of endothelial function, including decreased flow and nitrate-mediated dilation, were reduced, emphasizing the link between chronic inflammation and vascular impairment in this population.

Carotid IMT and pulse wave velocity are non-invasive methods to assess subclinical atherosclerosis and arterial stiffness. Pulse wave velocity is inversely related to arterial distensibility and is defined as the velocity of the arterial pulse moving along the vessel wall [105]. Of note, these tests do not come without limitations. Pulse wave velocity may not be significantly affected in early aortic atherosclerosis as the aorta can maintain its elasticity. Furthermore, particular vascular regions are more prone to atherosclerotic changes, such as the femoral artery when compared to the radial artery, these differences may affect the accuracy when measuring overall arterial stiffness [86].

Whether these results correlate with the future occurrence of CVD or cerebrovascular accidents in patients with SjD is still unclear. Bartoloni et al. found a statistically significant increased risk of myocardial infarction and cerebrovascular accidents in patients with SjD [35]. Increased age and disease duration directly correlated with an increased number of total cardiovascular events. Zippel et al. also found increased risk of stroke in SjD with cerebrovascular events occurring at an average age of 55.2 years [106]. However, of note, other studies have failed to make this statistically significant correlation. For example, Chiang et al. conducted a large nationwide longitudinal cohort study of patients newly diagnosed with SjD with no prior history of stroke and followed them until the end of the study time period or until they developed an ischemic stroke or until death. In this analysis, the incidence of ischemic stroke and myocardial infarction were similar between SjD patients and the control group [36,107,108]. A cross-sectional analysis of The Healthcare Cost and Utilization Project administrative longitudinal database from Luni et al. found a slightly lower risk for ischemic heart disease in SjD compared to age- and sex-matched controls [109]. These mixed findings suggest that while subclinical changes are evident in SjD, their translation to overt cardiovascular events may be influenced by other factors.

**Figure 1:**
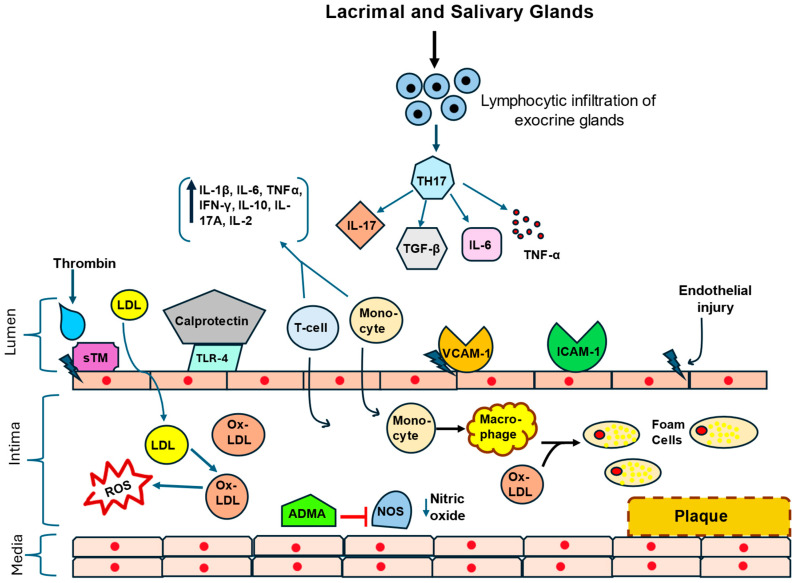
Overview of inflammatory events that promote the development and progression of atherosclerosis in the setting of SjD. This figure outlines potential inflammatory pathways that incite endothelial activation and atherosclerotic plaque formation in primary SjD. Production of adhesions molecules such as ICAM-1 and VCAM-1 accelerate endothelial injury by facilitating immune cell migration and penetration of the vessel wall. Monocytes that have migrated to the subintima differentiate into macrophages and take up ox-LDL to transform into foam cells. Ox-LDL is a major inducer of ROS from both endothelium and macrophages. In patients with SjD, lymphocytic invasion of salivary glands leads to glandular dysfunction and elevated circulating levels of the pro-atherogenic cytokine IL-17. Adding to the atheroma-promoting environment, activated epithelial cells within glandular tissue produce and secrete other key mediators of the immune response that propagate chronic inflammation such as IL-7, IL-18, and IL-22. Similarly, calprotectin, a key pro-inflammatory mediator of the innate immune response associated with both Sjögren’s disease and atherosclerosis, activates TLR-4. TLR-4, in turn, causes endothelial injury and the production of other key inflammatory markers such as IL-1β, IL-6, TNF-α, IFN-γ, IL-10, IL-17A, and IL-22. Plasma ADMA levels are elevated in SjD and can strongly inhibit nitric oxide synthase, increasing oxidative stress. The glycoprotein sTM, which is elevated in SjD, is activated by thrombin, regulates fibrinolysis and coagulation and its level is positively correlated with atherosclerotic risk. ADMA: plasma asymmetric dimethylarginine; ICAM-1: intracellular adhesion molecule-1; IL: interleukin; IFN-γ: interferon-gamma; LDL: low-density lipoprotein; NOS: nitric oxide sythase; ox-LDL: oxidized low-density lipoprotein; ROS: reactive oxygen species; sTM: soluble thrombomodulin; TLR-4: toll-like receptor-4; TNF-α: tumor necrosis factor-alpha; VCAM-1: vascular adhesion molecule-1.

Figure 1 depicts some of the major events and pathways involved in atherosclerosis in the inflammatory environment of SjD.

## 4. Surveillance and Diagnostics

### 4.1. Ultrasound Studies

Ultrasound is a valuable tool that can aid in diagnosing and assessing complications in SjD. It provides an efficient method to assess the involvement of the salivary gland in primary SjD, which is particularly useful given the variability and subjectivity of clinical symptoms [110]. Diagnosis relies on a combination of laboratory findings, such as positive auto-antibodies, clinical symptoms, and, occasionally, salivary gland biopsy. In 2016, the American–European consensus group criteria were revised and renamed to the ACR/EULAR classification criteria, which emphasizes objective disease measures but lacks imaging techniques. Studies have shown however that incorporating salivary gland ultrasound into the ACR/EULAR classification criteria can lead to improved performance of classification criteria fulfillment [111,112]. Ultrasound of the salivary glands can detect hypoechoic areas and increased heterogeneity within glandular tissues which signal inflammatory processes.

Additionally, in patients with SjD, echocardiography may serve as a useful tool to detect cardiac involvement. A 2008 study of 219 subjects (117 with primary Sjogren’s and 112 healthy controls) investigated whether echocardiographic abnormalities were associated with SjD in the absence of overt cardiac disease. Comprehensive echocardiographic examination found that, compared to controls, in the Sjogren’s cohort, mild mitral valve regurgitation, mild aortic valve regurgitation and tricuspid valve regurgitation were significantly more frequent. SjD patients were also more likely to have a mild pericardial effusion and more likely to have pulmonary hypertension. The Sjogren’s group also had a higher average left ventricular mass index [113]. Supporting these findings, a retrospective study by Ye et al. analyzed 124 SjD patients [114]. The authors found pericardial effusion in 20.2%, left ventricular diastolic dysfunction in 13.7% and pulmonary hypertension in 12.9%, reinforcing the relatively high prevalence of cardiac involvement in this population.

In addition to focusing on the heart, echocardiography can also be utilized to study the vasculature. In a 2024 study, researchers investigated the extent of subclinical atherosclerosis in patients with primary SjD utilizing carotid ultrasound to measure carotid IMT [15]. Subclinical atherosclerosis in Sjogren’s was defined through increased carotid IMT and presence of plaque. Doppler ultrasound measurement revealed that the primary SjD group had a higher IMT compared to the healthy control group (*p* < 0.0001).

These studies highlight the utility of ultrasound modalities in assessing cardiovascular abnormalities associated with SjD. When combined with laboratory data measuring lipid profile and inflammatory markers, ultrasound may provide a more comprehensive approach to risk stratification in these patients. Future studies should explore the utility of incorporating ultrasound into clinical guidelines as a routine assessment tool to evaluate for cardiovascular complications in SjD patients.

### 4.2. Other Imaging

While ultrasound can provide valuable information into the structural pathology of the heart, other imaging modalities can supplement ultrasound in the assessment of CVD in patients with SjD. For instance, cardiac magnetic resonance imaging (MRI) is considered the gold standard when evaluating for systemic heart disease [115,116]. Its ability to detect myocardial fibrosis can be invaluable in the assessment of cardiac scarring as a result of autoimmune diseases such as SjD [117].

A cross-sectional study in 2021 evaluated myocardial fibrosis in SjD patients through cardiac MRI [118]. Myocardial fibrosis was illustrated through the presence of late gadolinium enhancement. Among the 52 women with SjD enrolled, 10 were found to have late gadolinium enhancement and a salivary gland focus score ≥ 3 was independently associated with late gadolinium enhancement-positivity. Additionally, 3 patients demonstrated high intensity signals on T2-weighted imaging. indicating the presence of edema, and often indicative of inflammation associated with rheumatic disease [119].

The findings of late gadolinium enhancement and T2-weighted imaging are not uncommon in SjD patients. A pilot study involving 37 SjD patients also identified late gadolinium enhancement in 11 subjects using cardiac MRI. Researchers also found that Raynaud’s phenomenon was associated significantly with positive late gadolinium enhancement (*p* = 0.001) and T2-weighted imaging (*p* = 0.04) findings. Similarly to the Nishiwaki study, a salivary gland focus score above 3 was associated with positive late gadolinium enhancement findings of cardiac MRI, underscoring the connection between systemic autoimmune activity and subclinical myocardial involvement [120].

Apart from myocardial fibrosis, other conditions like that of autoimmune induced myocarditis are also evaluated through cardiac MRI. In a 2021 case report, a female, age 69 years with a history of SjD presented with symptoms of palpitations, chest pain and dyspnea on exertion for several weeks [121]. Electrocardiography revealed ST elevations and a right bundle branch block, while left heart catheterization revealed non-obstructive coronary artery disease. Cardiac MRI showed a reduced ejection fraction of 20% and late gadolinium enhancement, leading to a diagnosis of autoimmune myocarditis secondary to SjD. The patient was treated with corticosteroids, but ultimately passed away due to renal, cardiac, and hemodynamic complications.

Yokoe et al. utilized feature tracking cardiac MRI to evaluate for subclinical left ventricular dysfunction in SjD patients [122]. In this cross-sectional study, myocardial dysfunction measured via non-contrast-enhanced cardiac MRI in 50 female patients with Sjogren’s disease revealed lower longitudinal and circumferential strain measurements in SjD patients without cardiac symptoms, indicating subclinical heart disease.

Cardiac involvement in SjD is not just limited to subclinical findings. In a 2016 case report by Llanos-Chea, a 74-year-old female presented with shoulder pain, dyspnea on exertion, and worsening fatigue [123]. She was found to have an ejection fraction of 20% on echocardiography, with laboratory data showing an anti-nuclear antibody titer of 1:80, SSA > 8.0, SSB = 2.2, and an RF of 74 IU/mL. On cardiac MRI, she had findings of late gadolinium enhancement in the inferolateral wall and transmural basal-inferior segment. She was diagnosed with acute myocarditis, likely exacerbated by her longstanding SjD. Treatment with glucocorticoids resulted in improved systolic and diastolic function on repeat echocardiograms. This case underscores the utility of cardiac MRI, amongst other tools, in detecting autoimmune-mediated cardiac involvement and guiding targeted therapy.

Although computed tomography (CT) is not generally used for evaluation of coronary disease in SjD, it has proven to be valuable in detecting pericardial thickening and pericardial effusions related to SjD-associated pericarditis [124,125]. Moreover, the increased risk of aortic aneurysms and dissection amongst patients with SjD further highlights the value of CT in surveillance, but no study has evaluated the impact of CT-based surveillance on clinical outcomes or mortality in this population [126]

### 4.3. Blood Testing

The presence of subclinical cardiac involvement in SjD raises the question of whether patients without overt cardiac manifestations should undergo routine screening. While modalities such as cardiac MRI and echocardiography are costly, laboratory data might serve as an effective initial diagnostic tool. From prior studies, clinicians may consider utilizing lab measures of triglycerides, inflammatory markers, and even calprotectin to assess CVD risk in patients with SjD.

In a 2013 study by Juarez et al., traditional cardiovascular risk factors were compared between 543 SjD patients and 473 controls [127]. In this predominantly female cohort, patients with SjD had a higher prevalence of hypertriglyceridemia (21% vs. 9.5% *p* = 0.002) and hypertension (28–50% vs. 15.5–25.6%, *p* < 0.01). Of the SjD patients, 7 total cardiovascular events were recorded, 2 of which occurred in patients with hypertriglyceridemia and 1 in a patient with hypercholesterolemia (LDL). A retrospective study of 102 patients with a 20-year follow-up analyzed the prevalence of cardiovascular risk factors and their association with extraglandular involvement and antibody positivity [128]. Hypertension was present in 59% of patients, with a significantly higher prevalence among those with extraglandular involvement (*p* = 0.04). Compared to patients with glandular involvement alone, extraglandular involvement was associated with a greater frequency of dyslipidemia, higher mean LDL, and a higher prevalence of coronary disease. Multivariate analysis identified hypergammaglobulinemia, elevated ESR, and low C3 levels as independent predictors of increased cardiovascular risk. Patients receiving hydroxychloroquine were also noted to have a lower frequency of coronary artery disease, a finding supported by a study from Taiwan demonstrating reduced CVD risk in SjD patients being treated with hydroxychloroquine [129].

Calprotectin, an acute phase protein that is elevated in chronic inflammatory states, has emerged as a potential biomarker of carotid atherosclerosis in SjD [130]. A study of cardiovascular risk factors for atherosclerosis in 63 SjD patients and 63 age- and sex-matched controls used carotid ultrasound to assess the presence of atherosclerotic plaque. The study found that in the SjD group, there was a higher prevalence of carotid atherosclerosis (13% vs. 2% *p* = 0.015) and that calprotectin was associated with the presence of carotid atherosclerosis, independent of traditional risk factors. A bioinformatic study found calprotectin to be a hub gene in a common network for the pathogenesis of myocardial infarction and SjD, further supporting these findings [131].

A 2010 case–control study evaluated the prevalence of traditional cardiovascular risk factors (defined as age, hypertension, smoking, diabetes, obesity, hypercholesterolaemia, hypertriglyceridemia, and HDL levels) in SjD patients. A total of 312 SjD were enrolled and matched against 312 controls [73]. The study found that patients with SjD had a 1.5-fold higher prevalence of hypertriglyceridemia compared to controls. Corticosteroid treatment was associated with a higher frequency of hypertriglyceridemia and hypertension in the SjD group. While this study evaluated SjD in the context of defined cardiovascular risk factors, in 2024, Yang et al. investigated the lipid profiles of SjD patients in China in a retrospective study and found that 48.8% of patients had dyslipidemia [63]. Male patients were found to have significantly higher total cholesterol levels and LDL cholesterol. Additionally, increased serum triglyceride level was correlated with heightened disease activity (measured by ESSDAI scores of low, medium and high) (*p* = 0.003). Interestingly, patients with positive anti-SSA/SSB antibodies were found to have lower concentrations of total cholesterol, HDL, and LDL cholesterol. The study also found that elevated levels of CRP was a risk factor for hypertension and coronary heart disease, highlighting the interplay among dyslipidemia, inflammation, and autoimmunity.

Proprotein convertase subtilisin/kexin type 9 (PCSK 9) is a protein that promotes the degradation of LDL receptors thereby leading to regulation of lipid metabolism. In a 2023 cross-sectional study, researchers studied the association between PSCK9 levels, markers of atherosclerosis and autoimmune disease activity in 52 patients with SjD compared to 26 healthy controls [132]. The study found that compared to the control group, the SjD group had higher median plasma PCSK9 levels (162 vs. 53), higher prevalence of subclinical atherosclerosis (characterized by aortic pulse wave velocity measurements) but lower lipid levels. Notably, SjD patients exhibited a 3-fold increase in PCSK9 levels, despite having a lower prevalence of dyslipidemia and a higher cardiovascular risk profile compared to the control group.

These findings suggest that certain laboratory values and biomarkers may be helpful in detecting subclinical CVD or for supplementing traditional cardiovascular risk factors in the assessment of SjD patients. Elevated serum triglyceride levels appear to be associated with increased SjD disease activity, while calprotectin is associated with atherosclerotic risk and CRP elevation. Validation of these biomarkers is essential before incorporating them into routine clinical screening protocols.

Table 2 summarizes our current modalities for diagnosing and monitoring the cardiovascular system in persons with SjD.

### 4.4. Validity of Risk Assessment Tools

Cardiovascular risk assessment tools, such as the Framingham score, tend to underestimate cardiovascular risk in autoimmune disease, such as RA and SLE. [133,134,135,136]. Modified versions of these risk assessment tools such as the European Systematic Coronary Risk Evaluation algorithm (SCORE) calculator have been studied in many autoimmune diseases, but never in SjD [137,138]. In light of the multiple studies supporting the increased risk of CVD in SjD, it is safe to assume that these tools are inaccurate in SjD patients as well. Moreover, EULAR recommendations continue to endorse the use of these tools (such as Framingham score and SCORE) in patients with rheumatic disease, as well as an aggressive assessment of risk factors, further highlighting the need for further investigation of a predictive model that incorporates risk factors specific to SjD [139].

A 2024 study aimed to establish a risk prediction model of coronary heart disease in SjD based on IL-6 levels and regulatory T cell percentages [140]. Researchers collected data from 120 SjD patients in China, divided into those with and without coronary heart disease. They then gathered peripheral blood lymphocyte subsets and cytokine levels from both groups. Ultimately, this study identified ESR (OR 1.10, *p* = 0.019), triglyceride levels (OR 3.67, *p* = 0.041), IL-6 (OR 1,29, *p* = 0.048) and regulatory T cell percentage (OR = 0.25, *p* = 0.004) as independent risk factors for coronary heart disease in SjD patients.

Kintrilis et al. found that in SjD patients, the presence of arterial wall thickening, an indication of subclinical atherosclerosis, was more likely in those with elevated ESR levels and higher rates of familial CVD [141]. Additionally, advanced age and symptoms of dry eyes were associated with the presence of plaque. The study also found that in SjD patients, the presence of an AT genotype of B-cell activating factor (BAFF) led to higher risk of plaque formation and TATTT and TTCTT haplotypes were associated with increased susceptibility for arterial wall thickening. Building on these findings, Pu et al. developed the first nomogram to predict cardiovascular risk in SjD patients [142]. The model incorporated sex, initial symptom of joint pain, dry mouth, oral ulcers, dental caries, Raynaud’s phenomenon, fatigue, diabetes, elevated thyroid stimulating hormone, and systolic blood pressure as contributing factors to elevated cardiovascular risk.

## 5. Approaches to Treatment

### 5.1. Diet

Diet has been postulated to play a role in CVD in SjD, but there is limited data available regarding the role of diet in Sjogren’s related CVD. A cross-sectional study of SjD patient in Southern Italy found an inverse relationship between adherence to the Mediterranean diet and disease activity [143]. In addition, patients who reported consuming more fish had lower rates of hypertension. The authors suggest that the Mediterranean diet is beneficial for cardiovascular health in patients with SjD because it can reduce inflammatory burden. Ample fish intake may lower blood pressure as well.

However, not all studies have illustrated such clear associations. Another cross-sectional study of SjD patients found that adherence to the Mediterranean diet did not differ based on CVD risk stratification [144]. However, elevated serum uric acid was observed in the higher cardiovascular risk group, suggesting the possibility that CVD in SjD is related to hyperuricemia. This relationship may be mediated by uric acid-incited elevation of blood pressure [145,146]. Another cross-sectional study of 114 patients with SJD in France found that adherence to a Mediterranean diet was associated with less ocular dryness, and the authors propose that the reduced ocular symptoms may be attributed to the omega-3 fatty acid, antioxidants, and anti-inflammatory content of this type of diet [147].

A cross-sectional study from Karataş et al. used food consumption records to examine dietary intake patterns and their relationship to disease activity in 102 female SjD patients at a single center in Turkey [148]. Food records collected over 3 consecutive days were used to observe dietary intake trends and subjects were classified as consuming a pro-inflammatory or anti-inflammatory diet. Biochemical data including serum protein, albumin, triglycerides, total cholesterol, HDL cholesterol, LDL cholesterol, and CRP were obtained. The inflammatory content of the diet did not correlate with ESSDAI scores and the levels of ESR and CRP were not significantly different between the two subgroups. However, patients consuming a more pro-inflammatory diet had higher levels of total cholesterol, LDL, triglycerides, C3 and C4. These findings highlight the complexity of the effects of diet on both disease activity and cardiovascular risk in SjD, as well as the need for larger studies to clarify causal relationships.

Emerging evidence suggests that the gut microbiome may play a role in the relationship between autoimmune disease and CVD. Dysbiosis has been linked to both autoimmunity and CVD [149,150]. The production of short chain fatty acid metabolites from dietary fiber by a healthy microbiome can be atheroprotective [151]. The gut microbiome may be deregulated in the SjD population [152,153,154]. However, the connection between this abnormality and heart disease is, at present, an area of speculation based on findings in other diseases and in animal models [155]. Positive results of prebiotics supplementation have been reported in RA patients, to help improve RA clinical disease activity, but studies are needed to determine whether this would be true in SjD [156,157].

Clinical studies have demonstrated the benefits of a plant-based diet to overall cardiovascular health [158,159]. Adherence to a plant-based diet has shown positive results in a case series of three patients with SLE and SjD, with nearly all symptoms resolving in four weeks [160]. However, these are case reports that cannot be generalized. They suggest a direction for further exploration of dietary approaches in future studies without providing any significant evidence to support such interventions.

Intermittent fasting is another dietary regimen that may improve heart health by reducing body weight and blood pressure and improving glucose control [161]. In a non-obese diabetic mouse model, intermittent fasting was protective against salivary gland inflammation [162,163]. No clinical trials have explored intermittent fasting as a dietary modification strategy in SjD, however some studies in other autoimmune conditions suggest improved disease control [164].

Taken together, these findings suggest that the gut microbiome and dietary habits may present promising, yet severely underexplored, avenues for reducing cardiovascular risk and modulating disease activity in SjD. Larger studies are needed to clarify whether or not these strategies can be translated into meaningful clinical benefit.

### 5.2. Statins—Pros and Cons

In addition to their lipid-lowering effects, the anti-inflammatory pleiotropic properties of statins may benefit patients with autoimmune conditions [165]. Statins have multiple effects on the immune system. They decrease MHC Class II expression, T cell activation, co-stimulation molecules, adhesion molecule expression, cellular migration and intracellular signaling pathways [166]. Through these mechanisms, statins have been postulated to exert immunomodulatory effects [167,168].

It is known that SjD increases the risk of cardiovascular mortality, making their use for cardioprotective effects in SjD plausible. There is extensive data on the use of statins for cardioprotection in autoimmune diseases, with literature derived from mixed cohorts of patients with various systemic autoimmune conditions [169,170]. A population-based cohort study in the UK examined the benefit of statins in premature CVD amongst patients with rheumatic disease [171]. Patients from an electronic database with systemic autoimmune rheumatic diseases were categorized based on whether or not they were taking statins. Notably the group consisted of a mix of patients with multiple rheumatologic conditions, with lupus being the leading diagnosis. Approximately 30% of the patients had a diagnosis of SjD, with 30.1% of these in the statin group and 28.7% in the non-statin group. A hazard ratio of 0.63 (95% CI 0.42–0.94) indicates a 37% reduced risk of mortality in the SjD group for those taking statins.

On the other hand, Petri et al. found that statins were not effective in reducing subclinical atherosclerosis in lupus patients [172]. Further, side effects of statins can be substantial and include myopathy, rhabdomyolysis, and hepatotoxicity [173,174]. Statins may also interfere with glycemic control and contribute to the development of type 2 diabetes [175,176].

Currently, EULAR recommends comprehensive evaluation for traditional cardiovascular risk factors and the same approach for management of hyperlipidemia in the SjD population as in the general population. [177]. However, further research is needed to determine whether adjusted treatment indications and targets are warranted in this unique patient population.

### 5.3. Hydroxychloroquine, Antiplatelet Therapy and Anti-Rheumatic Drugs

Data regarding the use of hydroxychloroquine for the prevention of coronary disease are mixed [178]. A cross-sectional study from a single center in China reported that the prevalence of atherosclerotic disease in SjD patients was 41.3% (64 out of 155 SjD patients) and the rate of major cardiovascular and cerebrovascular events was 5.2% [87]. Furthermore, the study also found that commonly used medications, such as hydroxychloroquine, cyclophosphamide, and glucocorticoids, had no impact on the presence or development of atherosclerosis.

A retrospective cohort study from Taiwan utilized the National Health Insurance Research Database to examine hydroxychloroquine use and the development of CVD in 1142 newly diagnosed SjD patients, stratified by medication compliance. Risk factors for CVD were adjusted and hazard ratio for development of CVD was calculated. Among patients aged 40–64 years, those with higher compliance and a high medication possession ratio had a lower risk of developing CVD compared with patients with lower compliance and a low medication possession ratio [129].

In patients with rheumatoid arthritis and diabetes, the use of hydroxychloroquine has been linked to modest reductions in HbA1c, suggesting improved glycemic control as a possible mechanism for lowering cardiovascular risk [179]. Furthermore, in RA patients, hydroxychloroquine has been linked to more favorable lipid profiles. A study conducted in Texas evaluated the association between hydroxychloroquine and lipid levels of RA patients [180]. In this cohort, 254 patients were treated with hydroxychloroquine and 1007 were not. Those being treated with the drug had favorable atherogenic lipid profile outcomes, with lower total cholesterol, triglycerides and LDL and higher HDL. Observational data in RA and SLE populations generally support these metabolic benefits. Despite these findings, there is a lack of high-quality evidence specifically addressing cardiovascular risk management in SjD and, unfortunately, standard SjD therapy has not shown effectiveness in protection against CVD [87]. As such, general principles for cardiovascular risk management remain in use, though patients with SjD should be considered at higher cardiovascular risk compared with individuals with similar traditional risk factors without the disease [181,182].

In a cohort study of 541 patients, including 312 with primary SjD and age- and sex-matched controls, individuals with SjD exhibited a higher prevalence of traditional cardiovascular risk factors, such as diabetes and hypertension. Importantly, patients treated with hydroxychloroquine experienced a lower incidence of cardiovascular disease, whereas those receiving corticosteroids had a higher incidence. These findings underscore the potential cardioprotective effects of hydroxychloroquine in SjD, as well as the well-known pro-atherogenic impact of corticosteroid therapy [73].

Patients with SjD, who also have traditional cardiovascular risk factors may benefit from the addition of antiplatelet therapy, if deemed appropriate based on individual risk profiles. However, no specific recommendations exist regarding aspirin use in SjD. Additionally, individuals with co-existing autoimmune conditions and high-risk coagulation profiles, such as those with antiphospholipid syndrome (APS), should be considered for antiplatelet therapy [183].

Clinical studies evaluating conventional synthetic DMARDs, including methotrexate, azathioprine, leflunomide, mycophenolate mofetil, and cyclosporine, have not demonstrated benefits in ocular or oral dryness in SjD. Evidence is also limited regarding the role of DMARDs, except for hydroxychloroquine, or biologic therapies in reducing cardiovascular risk in patients with SjD. Together, these findings emphasize that while general cardiovascular risk management principles apply, targeted studies are needed to define optimal strategies, including pharmacologic interventions, for reducing cardiovascular risk, specifically in the SjD population.

## 6. Conclusions

CVD has long been known as a leading cause of morbidity and mortality in many rheumatic disorders, but little is known about the effect of SjD given its indolent course and general lack of treatment options. It is generally thought that the chronic inflammatory state accelerates atherosclerosis and plays a large role in premature CVD and death within this patient population. The effects of medications, especially glucocorticoids, have been thought to further propagate this disease course. Currently, imaging and diagnostic lab testing plays a limited role in assessing cardiovascular risk and risk assessment tools tend to underestimate the risk of CVD in this patient population. Moreover, given the rarity of SjD, most studies have limitations, such as small sample size and confounding comorbidities. Further research is needed to improve CVD risk assessment and to develop strategies to prevent severe cardiac consequences in this high-risk population.

## Figures and Tables

**Table 1:** Relative Risk of CVD in SjD Patients and Risk Factors specific to CVD and SjD.

Risk Factors	Findings in the Literature	References
Relative risk of CVD in SjD patients	CVD risk in SjD not as well established as in SLE and RA in the literature Mortality in SjD patients higher compared to the general population with CVD as the third leading cause of death. Hypertension, hypercholesterolemia, cerebrovascular events, and myocardial infarction more common in SjD Statistically significant association between decreased ABI and disease duration in SjD SjD patients have a higher carotid and femoral artery IMT Strong genetic link between SjD and CVD	[16,17,18,19,20,21,22,23,24,25,26,27,28,29,30,31,32,33,34,35,36,37,38,39,40,41,42]
Traditional CVD risk factors	Triglyceride levels higher in obese patients with SjD compared to non-obese SjD, however anti-SSA/SSB antibodies and RF were lower in obese population possibly suggesting protective effects of obesity Statins exhibit anti-inflammatory effects and are associated with a lower ESSDAI score Hypertension has the highest incidence in chronic inflammatory conditions Smoking associated with lower HDL levels in SjD patients however further research is warranted as studies currently show a variable or negative association between smoking and SjD	[43,44,45,46,47,48,49,50,51,52,53,54,55,56,57,58,59,60,61,62,63,64,65,66,67,68]
Risk factors specific to SjD	CRP not associated with increased IMT and does not differ between SjD vs. control SjD patients with higher carotid IMT show higher prevalence of leukopenia and circulating anti-SSA antibodies	[38,39,69,70,71,72,73,74,75,76,77,78]

Abbreviations: CVD = cardiovascular disease; SLE = systemic lupus erythematosus; RA = rheumatoid arthritis; IMT = intima-media thickness; ABI = ankle-brachial-index; RF = rheumatoid factor; SSA/SSB = Ro/La antigens; HDL = high-density lipoprotein; ESSDAI = EULAR-SS Disease Activity Index; CRP = C-reactive protein.

**Table 2:** Surveillance and diagnostic measures used for assessment of the cardiovascular system in persons with Primary Sjogren’s Disease.

Surveillance and Diagnostic Tool	Application	References
Ultrasound	Determine carotid intima-media thickness to assess subclinical atherosclerosis Pulse wave velocity to assess arterial stiffness Imaging of salivary glands which can indicate underlying inflammatory changes	[110,111,112,113,114]
Echocardiogram and cardiac magnetic resonance imaging	Increased incidence of valvular abnormalities, pericardial effusion, and pulmonary hypertension Myocardial fibrosis Subclinical left ventricular dysfunction	[115,116,117,118,119,120,121,122,123]
Lipid profile and blood biomarkers	Hypertriglyceridemia Elevated mean LDL Elevated calprotectin Elevated plasma PCSK9	[63,73,130,132]

## Data Availability

Not applicable.
